# Individual and Combined Effect of *MAO-A*/*MAO-B* Gene Variants and Adverse Childhood Experiences on the Severity of Major Depressive Disorder

**DOI:** 10.3390/bs13100795

**Published:** 2023-09-26

**Authors:** Christian Gabriel Toledo-Lozano, Luz Berenice López-Hernández, Juan Antonio Suárez-Cuenca, Luis Villalobos-Gallegos, Dulce Adeí Jiménez-Hernández, Sofía Lizeth Alcaraz-Estrada, Paul Mondragón-Terán, Lilia Joya-Laureano, Ramón Mauricio Coral-Vázquez, Silvia García

**Affiliations:** 1 Department of Clinical Research, Centro Médico Nacional “20 de Noviembre”, ISSSTE, Mexico City 03229, Mexico; christian.toledo@issste.gob.mx (C.G.T.-L.); suarej05@gmail.com (J.A.S.-C.); dulceadeichuche@gmail.com (D.A.J.-H.); 2 Life Cycle Department, Autonomous University of Guadalajara, Zapopan 45129, Mexico; luzb.lopez@edu.uag.mx; 3 Facultad de Medicina y Psicología, Universidad Autónoma de Baja California, Tijuana 22390, Mexico; villalobos.luis@uabc.edu.mx; 4 Department of Genomic Medicine, Centro Médico Nacional “20 de Noviembre”, ISSSTE, Mexico City 03229, Mexico; sofia.alcaraz@issste.gob.mx; 5 Coordination of Research, Centro Médico Nacional “20 de Noviembre”, ISSSTE, Mexico City 03229, Mexico; paul.mondragon@issste.gob.mx; 6 Department of Psychiatry and Psychology, Centro Médico Nacional “20 de Noviembre”, ISSSTE, Mexico City 03229, Mexico; liliajoya@yahoo.com.mx; 7 Department of Teaching and Research, Centro Médico Nacional “20 de Noviembre”, ISSSTE, Mexico City 03229, Mexico; rcoral@ipn.mx; 8 Postgraduate Section, Escuela Superior de Medicina, Instituto Politécnico Nacional, Mexico City 11340, Mexico

**Keywords:** *MAO-A*, *MAO-B*, major depressive disorder, gene-environment interaction, adverse childhood experiences

## Abstract

Background: Major depressive disorder (MDD) is a mood disorder with a high prevalence worldwide that causes disability and, in some cases, suicide. Although environmental factors play a crucial role in this disease, other biological factors may predispose individuals to MDD. Genetic and environmental factors influence mental disorders; therefore, a potential combined effect of *MAO-A*/*MAO-B* gene variants may be a target for the study of susceptibility to MDD. This study aimed to evaluate the effects of *MAO-A* and *-B* gene variants when combined with adverse childhood experiences (ACEs) on the susceptibility and severity of symptoms in MDD. Methods: A case-control study was performed, including 345 individuals, 175 MDD cases and 170 controls. Genotyping was performed using real-time PCR with hydrolysis probes. The analysis of the *rs1465107* and *rs1799836* gene variants of *MAO-A* and *-B*, respectively, was performed either alone or in combination with ACEs on the severity of depression, as determined through specific questionnaires, including DSM-IV diagnostic criteria for MDD. Results: According to individual effects, the presence of ACEs, as well as the allele G of the rs1465107 of *MAO-A*, is associated with a higher severity of depression, more significantly in females. Furthermore, the allele *rs1799836* G of *MAO-B* was associated with the severity of depression, even after being adjusted by gene variants and ACEs (IRR = 1.67, *p* = 0.01). In males, the allele *rs1799836* G of *MAO-B* was shown to interact with SNP with ACEs (IRR = 1.70, *p* < 0.001). According to combined effect analyses, the severity of depression was associated with ACEs when combined with either allele *rs1465107* of *MAO-A* or allele *rs17993836* of *MAO-B*, whereas SNP risk association was influenced by gender. Conclusions: The severity of depression is related to either individual or combined effects of temperamental traits and genetic susceptibility of specific genes such as *MAO-A* and *MAO-B*.

## 1. Introduction

Among psychiatric illnesses, major depressive disorder (MDD) is a highly prevalent condition that clinically manifests as persistent feelings of sadness, emptiness, irritability, anhedonia, reduced concentration, low energy, reduced self-esteem, alterations in appetite and sleep quality, loss of interest in formerly pleasant activities or even, presents with psychotic symptoms that can cause significant impairment in life quality and functioning of individuals [1,2]. Without optimal diagnosis and appropriate intervention, MDD causes disability to patients, resulting in lost workdays, distress to their families, and sometimes suicide [3,4]. Therefore, the need to gain insights into biological mechanisms and genetic variants involved in this devastating disease is of utmost importance.

The mechanisms for developing MDD are not fully understood, but it is generally accepted that genetic and environmental interactions are involved in the development of MDD [5]. A study by Fernandez et al. estimated the heritability of MDD between 28% and 44% [6], whereas other factors such as ethnicity, personal history, religion, presence of ethnic groups and economic income [7], and even the type of family account for the rest [8]. Both genetic and environmental factors should be considered in order to obtain insights into the origins of MDD [9,10].

With regard to biological factors that explain the etiology of MDD, the monoaminergic hypothesis is one of the most accepted, postulating that neurotransmitters, such as serotonin (5-HT), dopamine (DA), and norepinephrine (NE) levels are depleted. Nevertheless, it does not explain the delay in response to antidepressants and the lack of response in many patients, and it remains to be proven how such depletion occurs [11]. 5-HT, DA, and NE synthesis and degradation are implicated as a central mechanism in MDD. The degradation process of monoamines is presumably exacerbated in patients with MDD. Whereas the 5-HT, DA, and NE synthesis were proven to be decreased in depression disorders [12]. 

Monoamine oxidases (MAOs) are a family of enzymes encoded by genes located on the X chromosome. MAOs have oxidative catalytic activity on several neurotransmitters that work in the brain and surrounding tissues [13]. It has been suggested that MAOs are involved in the metabolism of primary, secondary, and tertiary amines ingested in food and the inactivation of several monoamine neurotransmitters [14]. MAO-A and MAO-B are the two isoenzymes of MAO with 70% amino acid identity and different affinity to their substrates. The MAO-A enzyme metabolizes mainly hydroxylated amines such as 5-HT, NE, and DA, while more hydrophobic amines such as benzylamine and phenylethylamine are mostly oxidized by MAO-B in the human brain and other tissues. Whereas MAO-B also has good affinity for 5-HT but not for NE and DA [15,16]. The oxidative deamination process of 5-HT, NE, and DA by MAOs is intimately associated with complex processes such as emotion modulation, motor activity, and cognitive functions [17,18]. Therefore, MAO activity has been associated with MDD and other conditions, such as autism-like and aggressive behaviors [19]. Disease-causing variants in MAO genes may affect the enzymatic activity and abundance of the enzymes and, consequently, a neurotransmitter imbalance. The *rs1465107* and *rs1799836* are variants in the *MAO-A* and *MAO-B* genes, respectively, which have been associated with MDD and other psychiatric disorders [11]. For instance, the *rs1465107* was associated with MDD in the Chinese population [20], whereas the *MAO-B rs1799836* variation was associated with higher MAO-B activity in several psychiatric conditions related to higher severity of alogia [21,22]. Additionally, researchers explored the *rs1799836* variant within the MAO-B gene in Mexican psychiatric patients who displayed symptoms of anhedonia [23]. Likewise, our group has a particular interest in *MAO-A* and *MAO-B* gene variants due to incipient results during standardization experiments.

Among environmental factors that contribute to MDD, adverse childhood experiences (ACE) are a significant risk factor for the development of MDD, as well as increased severity and earlier onset of the disease and a potential moderator of MDD in adulthood. ACEs represent a set of events that occur during the early stage of development, which includes the loss of parents or close relatives, any type of abuse, parental mental illness, or substance use, among others [24], and it is demonstrated that an external factor alters the brain homeostasis at different levels, including neurochemical balance, epigenetic, and cell biology. There is also evidence that ACEs are involved in significant structural and functional alterations in several central nervous system (CNS) structures, such as the hippocampus, amygdala, reward-related system, and prefrontal cortex. Recent studies have shown a strong association between ACEs and MDD [25] as one of the most important environmental factors related to depressive disorders. Previous studies suggest that ACEs are present in approximately 45% of psychiatric disorders with onset in childhood and 26–32% for those that had their onset during adulthood [26]. 

A comprehensive understanding of the interaction between genetic variants and adverse childhood experiences (ACEs) in susceptibility to the development of major depressive disorder (MDD) remains poorly understood. However, it is hypothesized that certain individuals may harbor genetic predispositions that make them more susceptible to the impacts of ACEs, while ACEs themselves may instigate genetic modifications that increase the likelihood of developing MDD.

In Mexico, few studies addressing biological factors that may predispose to MDD have been published [27,28,29], and most of them did not consider the adverse environment altogether with genetic factors. Hence, herein, we examined the individual and combined effect of *MAO-A*/*MAO-B* gene variants and adverse childhood experiences on the severity of MDD in a sample composed of Mexican-mestizo individuals.

## 2. Materials and Methods

### 2.1. Participants

The population of the study was consecutively recruited. Both male and female participants older than 18 years old were included. The case group accomplished a confirmed diagnosis of major depressive disorder (MDD) assessed by a trained psychiatrist using the Diagnostic and Statistical Manual of Mental Disorders, fourth edition, text revision (DSM IV-TR) criteria; and participants were also considered as Mexican-mestizos, according to the following criteria: (1) both Mexican born parents; (2) Spanish-derived last name; and (3) Mexican born ancestry dating back to the third generation [30]. Subjects with substance use disorder, psychotic, mania, schizoaffective disorders, or other significant neuropsychiatric conditions (e.g., autism spectrum disorders, intellectual disabilities, neurocognitive disorders, obsessive-compulsive disorders, trauma-related disorders, and stress factors) were excluded. The control group included healthy subjects with similar characteristics but without MDD, also assessed by the same trained psychiatrist.

### 2.2. Setting

All participants provided written informed consent before their inclusion in the study. This cross-sectional association study was conducted in the Centro Médico Nacional “20 de Noviembre” of the Instituto de Seguridad y Servicios Sociales de los Trabajadores del Estado (ISSSTE) in Mexico City. The study was approved by the Institute’s Human Research and Ethics Committees.

### 2.3. Clinical Assessment

The assessment of MDD was performed through a non-structured interview exploring socio-demographic (e.g., age, economic income, geographic origin, civil status, education level, and religion) and clinical data. ACE was also evaluated according to previous literature, including parental divorce, death of close family members, sexual abuse, addiction in family members, and psychological or physical violence. Finally, a specific questionnaire including DSM-IV diagnostic criteria for MDD (a 17-item Spanish version of the Hamilton Depression Rating Scale (HDRS)) was applied [31,32].

### 2.4. DNA Isolation and Genotyping

Genomic Deoxyribonucleic Acid (DNA) was isolated from peripheral blood using the dodecyltrimethylammonium bromide/hexadecyltrimethylammonium bromide (DTAB/CTAB) method to obtain DNA [33]. Genotyping was performed using real-time PCR using TaqMan probes (Hydrolysis probes, Applied Biosystems, Foster City, CA, USA). Real-time PCR was performed on a LightCycler 480 II (Roche Diagnostics GmbH, Basel, Switzerland); PCR reactions were performed according to the manufacturer’s instructions, whereas sequences used for the analysis of the *rs1465107* of *MAO-A C___8817699_20 (NM_000240.3)* and *C___8878790_10* for the *rs1799836 MAO-B* (*NM_000898.4*) were available upon request. Genetic information was analyzed at the Department of Genomic Medicine, Centro Médico Nacional “20 de Noviembre”, ISSSTE, Mexico City.

### 2.5. Statistical Analysis

The Hardy–Weinberg equilibrium (HWE) was estimated in both groups using the χ^2^ test (https://ihg.gsf.de/cgi-bin/hw/hwa1.pl, accessed on 9 May 2021). Taking into consideration that MAO genes are X-linked, we only tested HWE between female cases and controls. The population of the study met the HWE. Statistical power was calculated using the software “Quanto” v.1.2. To test the main hypothesis of the study, we conducted three subsequent models using Poisson regression models. In Model 1, all variables (age, ACE, *rs1465107,* and *rs1799836*) were included using additive terms; in Model 2, we added both interactive terms *rs1465107* × childhood adversities and *rs1799836* × ACE; in Model 3 a *rs1465107* × *rs1799836* interactive term was included. These models were estimated separately for male and female responses due to the difference in MAO SNPs’ distribution. To compare the steps, we conducted a likelihood ratio test (LRT), which follows a χ^2^ distribution; pseudo-R2 statistics (Nagelkerke, Cox & Snell and McFadden), Akaike information criterion (AIC) and Bayesian information criterion (BIC) were also estimated. An improvement in the steps was considered when a significant difference in the χ^2^ test (at *p* < 0.05), higher pseudo R2 statistics, and lower AIC and BIC were found. Statistical analysis was conducted using STATA v14.0 for the Student *t*-test, chi-square test (χ^2^), Poisson regression models, and likelihood ratio test. Statistical significance was considered when *p* < 0.05.

## 3. Results

The study included 349 subjects that met the eligibility criteria. Four of them were excluded due to incomplete genotyping. In total, 345 individuals, i.e., 175 MDD cases and 170 controls, were genotyped (demographic characteristics can be seen in Table 1). The Hardy–Weinberg equilibrium test showed that alleles and genotypes were distributed according to expected frequencies in both groups (data are provided in Table 2, and calculator used: https://gaow.github.io/genetic-analysis-software/h/hardy/ accessed on 20 June 2023. The specific patient database is not publicly available due to hospital confidentiality policies).

Genotype frequencies between groups and association tests are shown in Table 2. Of note, analysis was performed only in females since MAO is contained in the “X” chromosome.

No differences in genotype or allele frequencies were observed in any of the genetic variants analyzed, not even when separated by gender. Characteristics by gender and bivariate comparisons are shown in Table 3.

In order to start dissecting specific effects of gene/environment factors, the following analyses were performed: the percentage of depressed patients with genetic risk and ACEs for *MAO-A* were A/G = 57.14% (28/49 patients) and G/G = 56.89% (33/58 patients); whereas the risk genotypes for MAO-B were A/G = 50.0% (22/44 patients) and G/G = 61.9% (39/63 patients). In addition, the presence of ACE resulted in a general increased risk of depression severity (*p* < 0.001), regardless of sex.

Additionally, to further test the working hypothesis, several potential interactions were weighted, including (1) gene–gene interactions and (2) gene–environment interactions, through subsequent regression models. These analyses elicited weighting the effect of several variables (model 1: age, ACE, *rs1465107,* and *rs1799836*) on the basis of additive terms. Likewise, interactive terms (*rs1465107* × childhood adversities and *rs1799836* × ACE) were added in model 2. Finally, model 3 included *rs1465107* × *rs1799836* as interactive terms (Table 4). During such sub-analyses, the increase in HDRS mean scores was 43% in females and 83% in males (Model 1). Furthermore, the presence of ACE substantially increased the risk of depression severity along the three models, up to almost seven times higher, regardless of sex (Table 4 and Table 5).

Concomitantly, the SNP *rs1465107* (*MAO-A*) resulted in an increased risk for severity of depression, where the presence of a G allele conferred a significantly higher risk for severity of depression, specifically in females (IRR = 1.42, *p* < 0.001). On the other hand, the presence of SNP *rs1799836* (*MAO-B*) also resulted in an increased risk for severity of depression, but only when combined with ACE (Models 2 and 3), where the presence of the A allele in females conferred a trend for higher risk of depression.

When comparing the fit indices for the models, Model 2 was clearly better than Model 1, as every criterion was met. For the comparison between Model 2 and Model 3, the latter had a marginal improvement in Nagelkerke and Cox & Snell R2 statistics, and non-significant differences in the LRT were obtained, with the information criteria (AIC/BIC) favoring Model 2. 

## 4. Discussion

The present study investigated the effects of monoamine oxidase A (*MAO-A*) *rs1465107* and monoamine oxidase B (*MAO-B*) *rs1799836* gene variants, in conjunction with adverse childhood experiences (ACEs), on the susceptibility and severity of major depressive disorder (MDD).

Several genes have been claimed to play a pathophysiological role in MDD. Specific gene variants of biological pathways may affect MDD severity and response to therapy. For example, the *BDNF* gene variant *rs6265* has been significantly associated with the severity of MDD, particularly among naive-to-therapy depressed patients [34]. Likewise, specific *SLC6A4* gene polymorphism increased the probability of response to SSRI [35]. On the other hand, gene variants such as those coding for the catechol-O-methyltransferase (*COMT*), dopamine receptor 2 (*DRD2*) and/or selected *5-HTR* SNPs have shown contrasting findings regarding their association with MDD and response to antidepressants [36,37]. Interestingly, many effects of gene polymorphisms are independent of their protein expression, and the precise responsible mechanism is still unclear. 

However, this study explored the specific participation of *MAO-A* and *MAO-B* gene variants due to incipient results during standardization experiments from our group. In this regard, previous studies evidenced that interactions between *MAO-A* gene variants and ACE may predict antisocial behavior later in life [38]. However, few studies have focused on gene–environment interactions and whether they affect antisocial behavior or MDD in adulthood [9,39]. The *MAO-A* gene encodes an enzyme that metabolizes neurotransmitters such as serotonin and norepinephrine. Research has shown that individuals with certain variants of the *MAO-A* gene have lower levels of these neurotransmitters, which can increase their vulnerability to developing MDD. The role of ACEs in the development of MDD emerges because stressful or traumatic events that occur during childhood may be linked to an increased risk of developing MDD in adulthood. Our hypothesis proposes that genetic variants render individuals susceptible to neurotransmitter anomalies that may be triggered by ACEs. Interestingly, the most significant finding of the present study was that the interaction between *MAO-A* gene variants and ACEs significantly influenced the severity of MDD symptoms. This is supported by the report by Cicchetti et al. that found that adolescents who experienced severe ACE during childhood and concomitantly carried the *MAO-A*-uVNTR gene variant displayed higher severity of depression [40]. As an outcome variable, HDRS was used to detect MDD symptoms and their severity; thus, it is possible SNPs could modify the effect of childhood life experiences [41], not only in the severity of depression but also in the appearance of subthreshold depression symptoms.

Thus, these observations are consistent with the hypothesis that the interaction between *MAO-A* gene variants and ACE is a common mechanism driving the severity and/or susceptibility of different mental illnesses, including depression or externalizing mental disorders [42]. In particular, the present study found that the G allele in the *rs1465107* variant of *MAO-A* potentiates the effect of ACEs on the development of depression during adult life. This suggests that individuals with this allele who have experienced ACEs are more likely to develop severe MDD.

On the other hand, the presence of SNP *rs1799836* (*MAO-B*) increased the risk for severity of depression and the presence of anhedonia, but only when combined with ACE, where the A allele in females conferred a trend for a higher risk, but not the G allele. This last observation is not consistent with other studies [43,44,45]. The potential explanatory mechanism for the effect of *rs1799836* SNPs may be related to the association with high *MAO-B* platelet activity in increased persistence and decreased impulsivity [46]. Consistently, a sex-selective effect observed of SNP *rs1799836* (*MAO-B*) in females may be explained by the fact that MAO genes are linked to X-chromosomes; thus, there could be differential effects in the association of the main and interactive effects of *rs1465107* or *rs1799836* in the severity of depression [47].

Our data also show that the presence of single nucleotide polymorphism (SNP) *rs1799836* (*MAO-B*) increased the risk for severity of depression and anhedonia, but only when combined with ACEs. The *MAO-B rs1799836* gene variant modulates MAO-B transcription, consequently influencing protein translation and MAOB activity [48]. It is important to note that the present study was conducted in a Mexican-mestizo population, and it is possible that the results may differ in this population.

The present study had some limitations, including a small sample size and a lack of completely appropriate matched controls. Additionally, only a limited number of polymorphisms of *MAO-A* and *MAO-B* were tested, while it would have been interesting to test other neurotransmitters and gene variants such as *SLC6A4, COMT, BDNF, and 5-HTR*. Future studies with larger sample sizes and more comprehensive genetic testing are needed to confirm the findings of the present study. The findings reported herein have some implications for further research on MDD. Individuals with certain MAO-A gene variants and ACEs may be more likely to develop MDD or may present with more severe MDD and may benefit from early intervention and treatment with medications that target the MAO-A enzyme.

Furthermore, the findings suggest that personality traits may play a role in the interaction between *MAO-A* gene variants, ACEs, and MDD. Future studies should investigate this possibility.

Most of the evidence related to MAO SNPs is the result of studies conducted mostly in Caucasian and Asian individuals [49]. Genotypic differences between populations are widely recognized; thus, showing results from the Mexican mestizo population is the strength and novelty of this research.

Interaction between ACE × *MAO-A* SNPs has been previously conducted in adolescents [39,41]. To our knowledge, the present study is the first one to evaluate the impact of the interaction of ACE × *MAO-A* SNPs on MDD in adults. In addition, the results from the present study suggest that (1) sequelae of ACE might be long-lasting during the whole life course and (2) the protective effect of some *MAO-A* SNPs might be equally significant across the life cycle.

Finally, we aimed to assess associations among MDD, childhood adverse experiences, and genetic variants in the MAO genes. However, it was interesting that no associations were found when only MDD and the genetic variants were included in the models. This may be explained by different factors, such as the presence of novel variants that are unique to the Mexican-mestizo population that are not present in most commercially available DNA chips that detect common variants based on well-known populations (often Caucasians). In addition, only variants in autosomes are frequently studied, so gene variants in the X chromosome are often underrepresented in such studies [50].

These results should be interpreted with caution due to some limitations, such as (1) low sample size, (2) the lack of completely appropriate matched controls, (3) the reduced number of polymorphisms tested and (4) the way how ACEs were evaluated (binary category), may give rise to potential interpretation bias. This last point represents a relative limitation, since assessments of ACEs were performed by an experienced psychiatrist, then confident diagnosis was warrant; in addition, categorical expression of ACEs is reliable to estimate risk associations.

Future studies could add the measurement of personality traits, as well as several other candidate genes, to map the interaction between these factors in the occurrence and severity of depressive symptoms.

## 5. Conclusions

Our data suggests that the interaction between gene variants and adverse childhood experiences significantly influences the severity of symptoms in major depressive disorder. Specifically, the G allele of the *rs1465107* in the *MAO-A* gene potentiates the effect of ACE to develop depression during adult life. In addition, the *rs1799836* variant in the monoamine oxidase B (*MAO-B*) gene increased the risk for severity of depression and the presence of anhedonia, but only when combined with ACE. This study is the first to evaluate the impact of the interaction between ACE and genetic variants in the MAO genes on MDD in adults. Adverse childhood experiences that involve stress, abuse, or illness may change brain homeostasis at different levels, including neurochemical and epigenetic complex interactions. Thus, (1) having adverse childhood experiences is related to MDD during adult life; (2) the combination between *MAO-A* and *MAO-B* gene variants may ameliorate to a certain level the association of adverse childhood experiences [1] and depression; (3) adding interactive terms that are associated to higher correlations with the HDRS total score, implied a better model of depression severity; and (4) both genetic factors and temperamental traits could ameliorate the impact of adverse childhood experiences in the development of MDD in adulthood. Further evidence from longitudinal studies is warranted to understand the nature of these associations better.

## Figures and Tables

**Table 1:** Demographic characteristics of the study groups.

Variable	Cases *n* = 175		Controls *n* = 170		*p*
Gender	Male *n* = 54	Females *n* = 121	Males *n* = 86	Females *n* = 84	0.0001
	−30.8%	−69.2%	−50.6%	−49.4%	
Age (years old)	31.50 (18–62) *	35 (19–65) *	29.50 (18–65)	30 (18–65) *	0.08
Economic income (Mexican Pesos)	$6900 (0–60,000) *	$4000 (0–70,000) *	$7250 (0–50,000) *	$6000 (0–60,000) *	0.07
Geographic Origin	42 (77.8%)	79 (65.3%)	54 (62.8%)	57 (67.9%)	0.73
CDMX	1 (1.9%)	6 (5%)	4 (4.7%)	3 (3.6%)	
Edo. Mex.	11 (20.4%)	36 (29.8%)	28 (32.6%)	24 (28.6%)	
Other					
Civil status					0.44
Single	38 (70.4%)	70 (57.9%)	55 (64%)	52 (61.9%)	
Married	11 (20.4%)	27 (22.3%)	26 (30.2%)	18 (21.4%)	
Free union	3 (5.6%)	6 (5%)	1 (1.2%)	2 (2.4%)	
Separated	2 (3.7%)	4 (3.3%)	0 (0%)	3 (3.6%)	
Widow	0 (0%)	8 (6.6%)	1 (1.2%)	5 (6%)	
Divorced	0 (0%)	6 (5%)	3 (3.5%)	4 (4.8%)	
Education					0.52
Elementary school	2 (3.7%)	5 (4.1%)	3 (3.5%)	3 (3.6%)	
Junior High	3 (5.6%)	10 (8.3%)	8 (9.3%)	8 (9.5%)	
High school	19 (35.2%)	43 (35.5%)	30 (34.9%)	17 (20.2%)	
Bachelor´s degree	15 (27.8%)	52 (43%)	29 (33.7%)	39 (46.4%)	
Postgraduate studies	15 (27.8%)	11 (9.1º%)	16 (18.6%)	17 (20.2%)	
Religion					0.07
Catholic	36 (66.7%)	70 (57.9%)	58 (67.4%)	63 (75%)	
Christian	1(1.9%)	12 (9.9%)	3 (3.5%)	2(2.4%)	
Jewish	1 (1.9%)	0 (0%)	1 (1.2%)	0 (0%)	
Agnostic	13 (24.1%)	31 (25.6%)	16 (18.6%)	13 (15.5%)	
Atheist	3 (5.6%)	8 (6.6%)	8 (9.3%)	6 (7.1%)	

* (minimum and maximum). CDMX: Ciudad de México; Edo. Mex.: Estado de México.

**Table 2:** Genotype frequencies and chi-square association test.

Gene Variant	Genotype (%)			Allele Frequency		OR (95%CI)	*p*-Value
	AA	AG	GG	A	G		
*rs1465107*							
Cases	9 (7.4%)	63 (52.1%)	49 (40.5%)	0.33	0.66	OR = 0.716	*p* = 0.50951
Controls	10 (11.9%)	35 (41.6%)	39 (53.5%)	0.32	0.67	(0.265–1.935)	
	AA	AG	GG	A	G		
*rs1465107*							
Cases	13 (10.7%)	63 (52.1%)	45 (37.2%)	0.36	0.63	OR = 0.982	*p* = 0.97007
Controls	10 (11.9%)	40 (47.6%)	34 (40.5%)	0.35	0.64	(0.385–2.507)	

**Table 3:** Sample characteristics by gender and bivariate comparisons.

	Male *n* =140	Female *n* = 205	Total *n* = 345	Statistical Difference
	$\bar{x}$ (SD)	$\bar{x}$ (SD)	$\bar{x}$	
Age	33.50 (13.40)	36.91 (14.17)	35.52 (13.91)	t (343) = 2.241, *p* = 0.025
HDRS score	5.90 (5.24)	7.80 (6.26)	7.13 (6.13)	t (343) = 2.948, *p* = 0.003
Monthly income (1000 MXP)	8.96 (9.83)	7.93 (10.82)	8.35 (10.42)	t (343) = 0.898, *p* = 0.369
	***n* (%)**	***n* (%)**	***n* (%)**	
Marital Status				χ^2^ (2) = 1.930, *p* = 0.381
Never married	93 (66.43)	122 (59.51)	215 (62.32)	
Married/Cohabiting	41 (29.29)	53 (25.85)	94 (27.25)	
Divorced/Widowed	6 (4.28)	30 (14.64)	36 (10.43)	
Education				χ^2^ (2) = 1.269, *p* = 0.530
Elementary	16 (11.43)	26 (12.68)	42 (12.17)	
High School	49 (35.00)	60 (29.27)	109 (31.59)	
College	75 (53.57)	119 (58.05)	194 (56.24)	
*rs1465107* SNPs ^b^				N/A ^a^
A/A	45 (32.14)	19 (9.27)	64 (18.55)	
A/G	0	98 (47.80)	98 (28.41)	
G/G	95 (67.86)	88 (42.93)	183 (53.04)	
*rs1799836* SNPs ^b^				N/A ^a^
A/A	47 (33.57)	23 (11.22)	70 (20.29)	
A/G	0	103 (50.24)	103 (29.86)	
G/G	93 (66.43)	79 (38.54)	172 (49.86)	
Childhood adversities (Yes)	65 (46.42)	107 (52.20)	172 (49.86)	χ^2^ (1) = 0.887, *p* = 0.346

Notes: ^a^ chi-squared test was not estimated, ^b^ SNPs: single-nucleotide polymorphism. HDRS: Hamilton Depression Rating Scale; MXP: Mexican Pesos, OR: Odds Ratio.

**Table 4:** Comparison of the regression models for the HDRS score in the women subsample (*n* = 205).

	Model 1 Additive Effects			Model 2 SNPs × ACE Effects			Model 3 SNPs × ACE and SNPs × SNPs Effects		
	IRR	95% CI	*p*	IRR	95% CI	*p*	IRR	95% CI	*p*
Intercept	3.34	2.50–4.47	<0.001	1.77	1.05–2.96	0.030	1.70	1.14–3.33	0.049
Age	1.00	1.00–1.00	0.003	1.00	1.00–1.00	0.018	1.00	1.00–1.00	0.010
rs1465107 (A/A)									
A/G	1.27	1.04–1.56	0.019	2.11	1.49–2.99	<0.001	1.95	1.14–3.33	0.014
G/G	1.42	1.16–1.74	0.001	1.98	1.39–2.80	<0.001	2.06	1.41–3.00	<0.001
rs1799836 (A/A)									
A/G	1.17	0.98–1.39	0.068	1.63	1.11–2.40	0.012	2.32	1.26–4.29	0.007
G/G	1.30	1.09–1.56	0.003	1.67	1.13–2.48	0.010	1.57	1.04–2.35	0.030
ACE	1.43	1.29–1.58	<0.001	3.91	2.19–7.00	<0.001	4.05	2.26–7.25	<0.001
ACE × rs1465107									
Yes × A/G	-	-		0.42	0.27–0.66	<0.001	0.42	0.28–0.65	<0.001
Yes × G/G	-	-		0.56	0.37–0.87	0.010	0.56	0.36–0.86	0.008
ACE × *rs1799836*									
Yes × A/G	-	-		0.64	0.42–0.99	0.048	0.64	0.41–0.99	0.044
Yes × G/G	-	-		0.73	0.47–1.13	0.164	0.73	0.47–1.14	0.162
*rs1465107* × *rs1799836* ^a^									
A/G × A/G	-	-		-	-		0.75	0.41–1.39	0.357
A/G × G/G	-	-		-	-		1.24	0.83–1.87	0.294
G/G × A/G	-	-		-	-		0.71	0.43–1.16	0.173
	**Estimate**			**Estimate**			**Estimate**		
AIC	1694.925			1675.436			1676.100		
BIC	1718.186			1711.989			1722.622		
Pseudo R^2^									
Nagelkerke	0.284			0.374			0.390		
McFadden	0.031			0.042			0.042		
Cox & Snell	0.284			0.374			0.390		
LRT	-			χ^2^ (4) = 27.49			χ^2^ (3) = 5.34		

*p* < 0.05. Data are shown as IRR, 95 IC, and *p*-value. Note: Comparison values are between parentheses. ^a^ the comparison category was G/G × G/G because the count of the combination of A/A × A/A is zero. Abbreviations: AIC = Akaike information criterion; LRT = likelihood ratio test; ACEs: adverse childhood experiences; SNPs: single-nucleotide polymorphism; BIC: Bayesian information criterion.

**Table 5:** Comparison of the regression models for HDRS score in the men subsample (*n* = 140).

	Model 1 Additive Effects			Model 2 SNPs × ACE Effects			Model 3 SNPs × ACE and SNPs × SNPs Effects		
	IRR	95% CI	*p*	IRR	95% CI	*p*	IRR	95% CI	*p*
Intercept	2.81	2.12–3.71	<0.001	1.57	1.07–2.29	0.020	1.67	1.07–2.60	0.025
Age	1.01	1.00–1.02	<0.001	1.01	1.00–1.01	<0.001	1.01	1.00–1.01	<0.001
*rs1465107* (A/A)									
G/G	0.90	0.78–1.04	0.163	1.27	0.99–1.63	0.059	1.19	0.82–1.71	0.357
*rs1799836* (A/A)									
G/G	1.13	0.97–1.31	0.100	1.70	1.31–2.20	<0.001	1.59	1.10–2.30	0.014
ACE	1.83	1.58–2.11	<0.001	4.17	2.90–5.98	<0.001	4.08	2.82–5.90	<0.001
ACE × *rs1465107*									
Yes × G/G	-	-		0.58	00.43–0.79	0.001	0.59	0.43–0.80	0.001
ACE × *rs1799836*									
Yes × G/G	-	-		0.53	0.38–0.72	<0.001	0.53	0.39–0.74	<0.001
*rs1465107* × *rs1799836*									
G/G × G/G	-	-		-	-		1.09	0.78–1.50	0.621
	**Estimate**			**Estimate**			**Estimate**		
AIC	959.085			937.449			939.206		
BIC	973.793			958.041			962.739		
Pseudo R^2^									
Nagelkerke	0.432			0.527			0.528		
McFadden	0.067			0.088			0.087		
Cox & Snell	0.432			0.527			0.528		
LRT	-			χ^2^ (2) = 25.64			χ^2^ (1) = 0.24		

*p* < 0.05. Note: Comparison values are in parentheses. Abbreviations: AIC = Akaike information criterion; LRT = likelihood ratio test; ACEs: adverse childhood experiences; SNPs: single-nucleotide polymorphism; BIC: Bayesian information criterion.

## Data Availability

Datasets analyzed or generated during this study can be requested from the authors.
